# Supplementary material for: Conditional effects of Cardinium on microbiota in an invasive whitefly under different ecological factors
Source: Microbiol Spectr. 2025 Aug 12;13(9):e02240-24. doi: 10.1128/spectrum.02240-24 (PMC12403816; doi:10.1128/spectrum.02240-24)
Supplement: Supplemental figures and table — Fig. S1 to S4 and Table S1. [file spectrum.02240-24-s0001.docx]

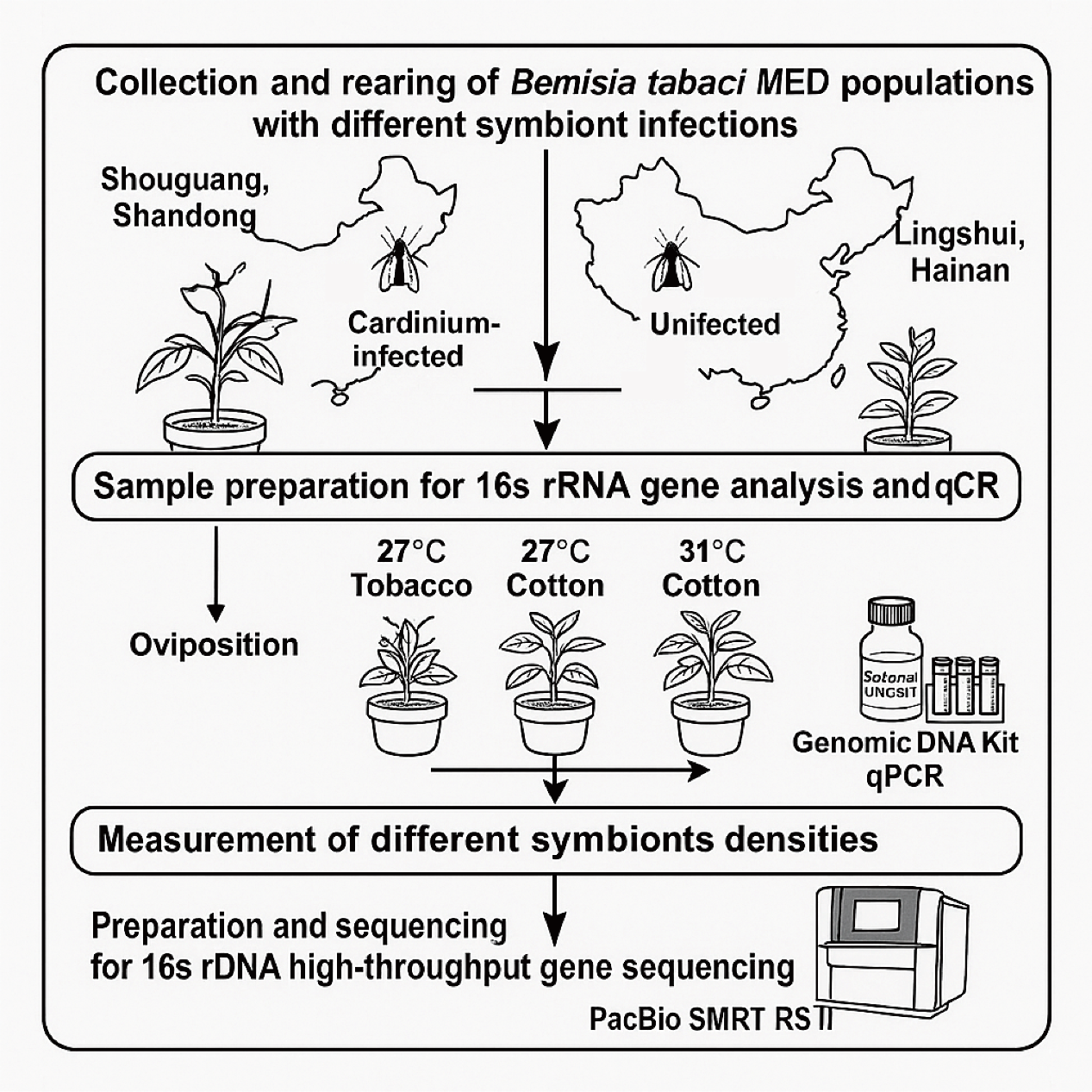
Fig. S1. Bemisia tabaci MED sample preparation for 16s rRNA gene analysis and qPCR experiment of LS and SG populations under different temperature and host plants.


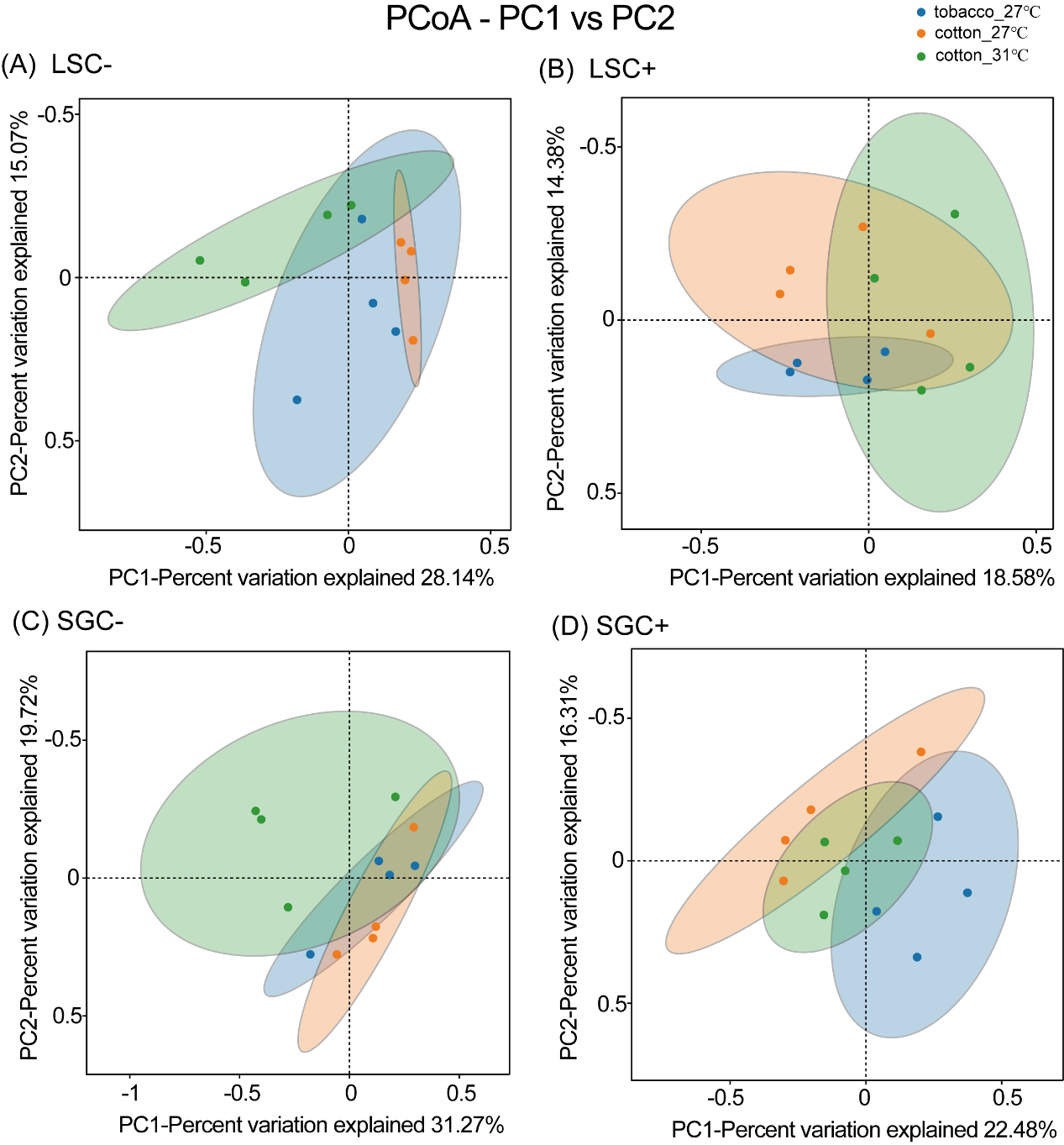


Fig. S2. Principal coordinates analysis of Bemisia tabaci MED of LS and SG populations under different temperature and host plants, LSC+ isofemale line (A), LSC- isofemale line (B), SGC+ isofemale line (C), and SGC- isofemale line (D) are shown, respectively.


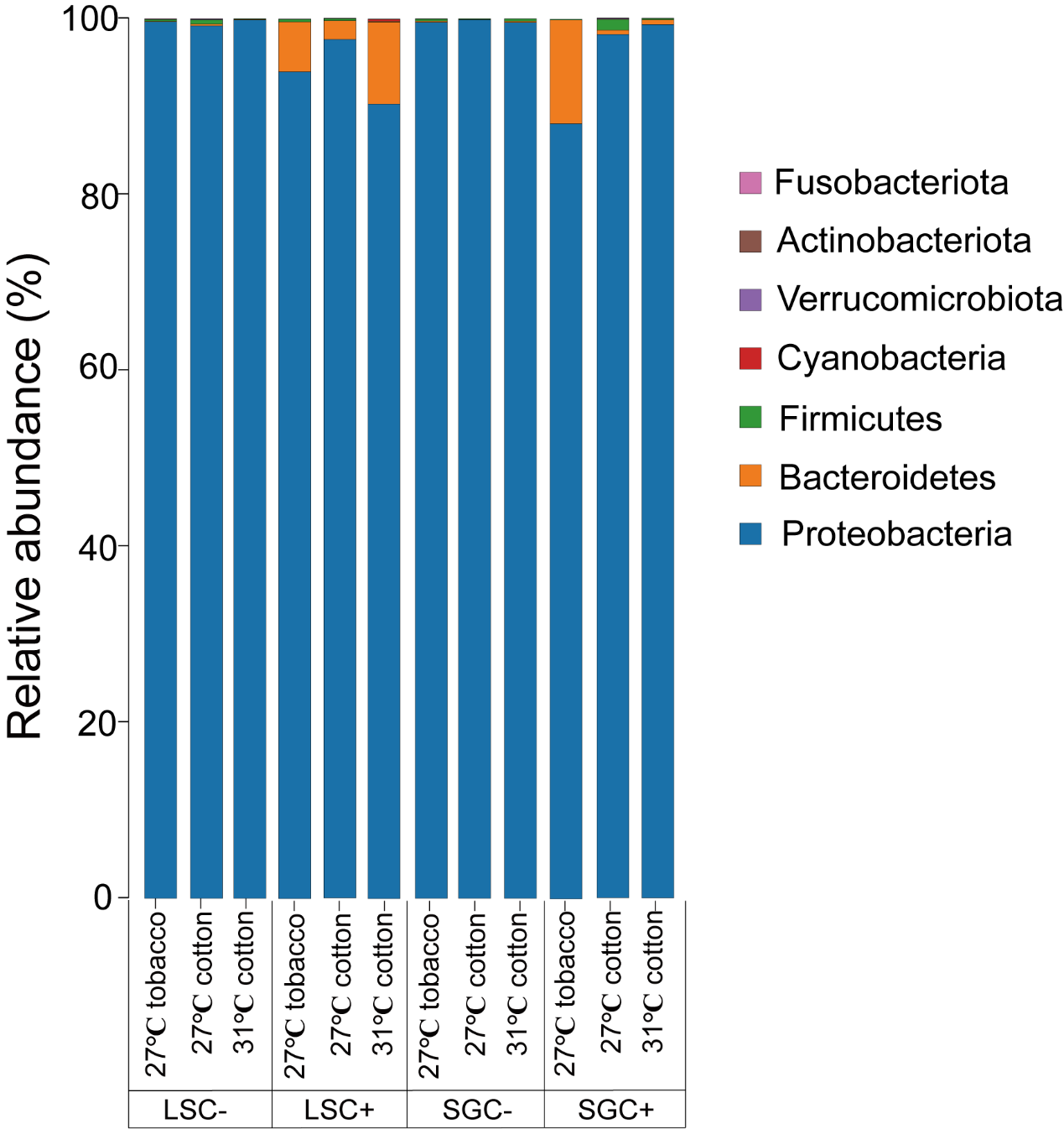


Fig. S3. Relative abundance of top 7 bacterial phylum in LS and SG population Bemisia tabaci MED under different treatment conditions, detected by full-length 16S rRNA gene sequencing.


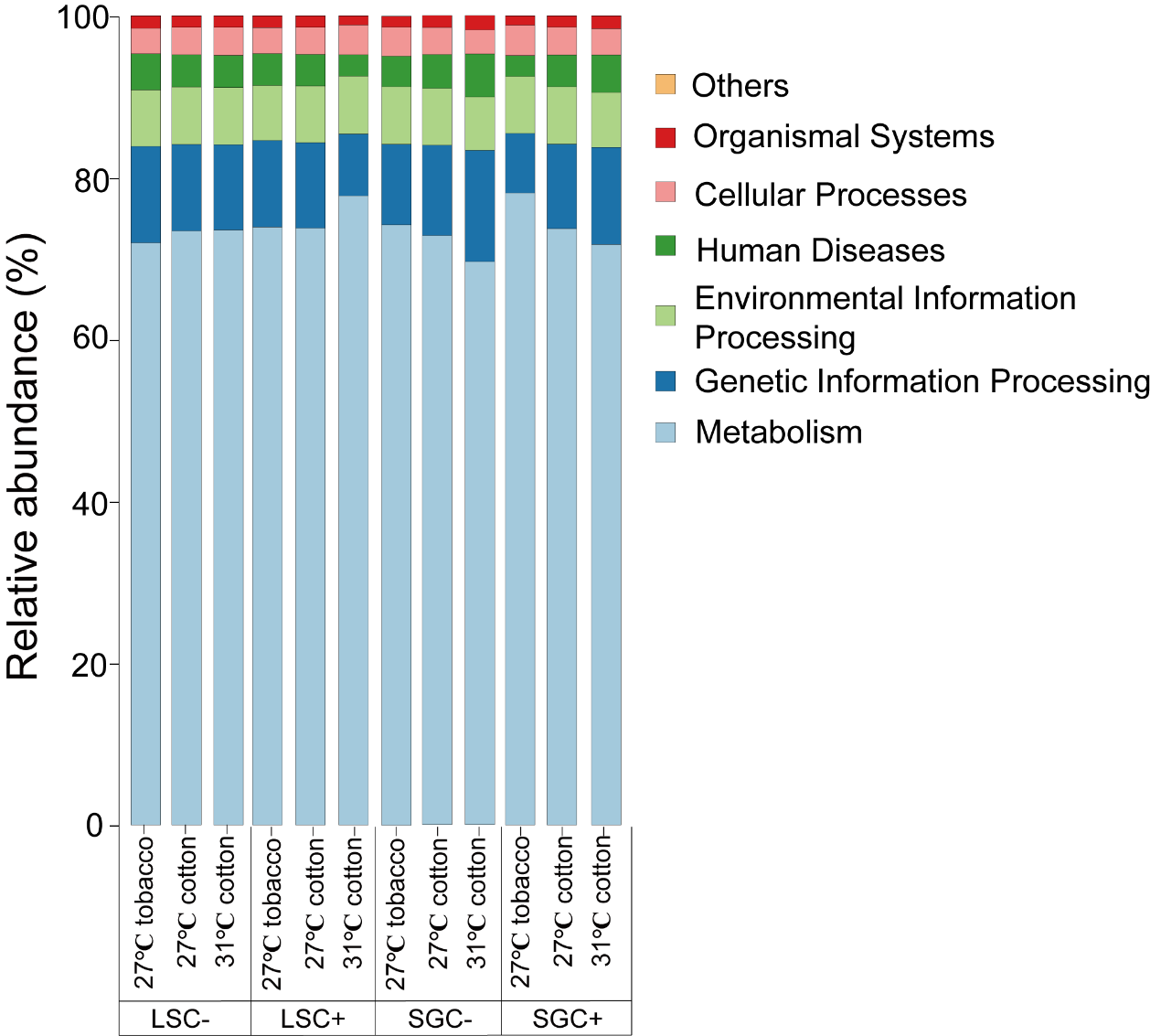


Fig. S4. Functional analysis of bacterial communities in LS and SD populations whiteflies grown under different temperature and host plant conditions based on PICRUSt (Phylogenetic Investigation of Communities by Reconstruction of Unobserved States) at BMK Cloud (https://www.biocloud.net).

**Table S1.** Sequencing results of full-length 16S rRNA gene of *Bemisia tabaci* LS and SG populations with different *Cardinium*-infected status in different host plants under various temperatures.

| Sample ID | Raw CCS | Clean CCS | Effective CCS | AvgLen(bp) | Effective(%) | Species |
| --- | --- | --- | --- | --- | --- | --- |
| SGC+_31cotton_1 | 12915 | 11670 | 11594 | 1436 | 89.77 | 8 |
| SGC+_31cotton_2 | 13135 | 11850 | 11781 | 1431 | 89.69 | 11 |
| SGC+_31cotton_3 | 13023 | 11699 | 11641 | 1432 | 89.39 | 12 |
| SGC+_31cotton_4 | 7921 | 7161 | 7104 | 1431 | 89.69 | 16 |
| SGC+_27cotton_1 | 12999 | 11650 | 11571 | 1442 | 89.01 | 15 |
| SGC+_27cotton_2 | 12937 | 11601 | 11525 | 1443 | 89.09 | 27 |
| SGC+_27cotton_3 | 12981 | 11709 | 11625 | 1444 | 89.55 | 13 |
| SGC+_27cotton_4 | 10499 | 9413 | 9350 | 1445 | 89.06 | 15 |
| SGC+_27tobacco_1 | 13007 | 11610 | 11532 | 1464 | 88.66 | 13 |
| SGC+_27tobacco_2 | 12955 | 11548 | 11464 | 1463 | 88.49 | 11 |
| SGC+_27tobacco_3 | 13007 | 11714 | 11611 | 1463 | 89.27 | 13 |
| SGC+_27tobacco_4 | 12982 | 11572 | 11462 | 1463 | 88.29 | 13 |
| SGC-_31cotton_1 | 8042 | 7282 | 7225 | 1426 | 89.84 | 7 |
| SGC-_31cotton_2 | 13053 | 11775 | 11700 | 1425 | 89.63 | 6 |
| SGC-_31cotton_3 | 13013 | 11654 | 11545 | 1431 | 88.72 | 19 |
| SGC-_31cotton_4 | 13024 | 11772 | 11704 | 1429 | 89.86 | 10 |
| SGC-_27cotton_1 | 12955 | 11642 | 11559 | 1442 | 89.22 | 17 |
| SGC-_27cotton_2 | 12946 | 11641 | 11559 | 1438 | 89.29 | 11 |
| SGC-_27cotton_2 | 13011 | 11740 | 11661 | 1438 | 89.62 | 21 |
| SGC-_27cotton_1 | 13038 | 11666 | 11575 | 1445 | 88.78 | 18 |
| SGC-_27tobacco_1 | 13043 | 11752 | 11646 | 1440 | 89.29 | 21 |
| SGC-_27tobacco_2 | 12675 | 11401 | 11289 | 1447 | 89.07 | 31 |
| SGC-_27tobacco_3 | 12879 | 11598 | 11515 | 1445 | 89.41 | 17 |
| SGC-_27tobacco_4 | 13052 | 11811 | 11705 | 1444 | 89.68 | 13 |
| LSC+_31cotton_1 | 12876 | 11507 | 11427 | 1466 | 88.75 | 19 |
| LSC+_31cotton_2 | 12974 | 11655 | 11574 | 1465 | 89.21 | 13 |
| LSC+_31cotton_3 | 10067 | 8969 | 8905 | 1465 | 88.46 | 14 |
| LSC+_31cotton_4 | 13033 | 11640 | 11538 | 1463 | 88.53 | 15 |
| LSC+_27cotton_1 | 10751 | 9655 | 9556 | 1445 | 88.88 | 24 |
| LSC+_27cotton_2 | 12471 | 11210 | 11102 | 1440 | 89.02 | 27 |
| LSC+_27cotton_3 | 13053 | 11758 | 11655 | 1452 | 89.29 | 13 |
| LSC+_27cotton_4 | 13036 | 11741 | 11656 | 1443 | 89.41 | 19 |
| LSC+_27tobacco_1 | 13002 | 11654 | 11560 | 1442 | 88.91 | 24 |
| LSC+_27tobacco_2 | 12963 | 11656 | 11562 | 1443 | 89.19 | 15 |
| LSC+_27tobacco_3 | 12979 | 11768 | 11675 | 1439 | 89.95 | 18 |
| LSC+_27tobacco_4 | 13005 | 11635 | 11541 | 1444 | 88.74 | 20 |
| LSC-_31cotton_1 | 13005 | 11667 | 11586 | 1443 | 89.09 | 6 |
| LSC-_31cotton_2 | 13106 | 11766 | 11686 | 1445 | 89.17 | 10 |
| LSC-_31cotton_3 | 13104 | 11865 | 11771 | 1439 | 89.83 | 18 |
| LSC-_31cotton_4 | 9872 | 8900 | 8833 | 1442 | 89.48 | 16 |
| LSC-_27cotton_1 | 8764 | 7847 | 7758 | 1444 | 88.52 | 25 |
| LSC-_27cotton_2 | 11660 | 10467 | 10334 | 1445 | 88.63 | 26 |
| LSC-_27cotton_3 | 9647 | 8697 | 8607 | 1447 | 89.22 | 24 |
| LSC-_27cotton_4 | 9947 | 8956 | 8875 | 1440 | 89.22 | 26 |
| LSC-_27tobacco_1 | 9254 | 8286 | 8210 | 1440 | 88.72 | 16 |
| LSC-_27tobacco_2 | 12939 | 11638 | 11552 | 1440 | 89.28 | 24 |
| LSC-_27tobacco_3 | 12965 | 11608 | 11526 | 1439 | 88.9 | 21 |
| LSC-_27tobacco_4 | 12922 | 11630 | 11547 | 1440 | 89.36 | 19 |

Raw CCS: all CCS (Circular Consensus Sequencing) sequence number in each sample identified by software ilma v1.7.0; Clean CCS: the number of CCS sequences after identifying and removing the primers by software [cutadapt 1.9.1](https://doi.org/10.14806/ej.17.1.200); Effective CCS: the number of CCS sequences after filtering the short sequences and chimeras by using software [UCHIME v4.2](http://drive5.com/uchime); Average Length (bp): the average length of all CCS sequences in this sample; Effective (%): the percentage of effective CCS in raw CCS; Species: the total number of bacterial species in this sample.
